# Dataset on inflammatory proteins expressions and sialic acid levels in apolipoprotein E-deficient mice with administration of N-acetylneuraminic acid and/or quercetin

**DOI:** 10.1016/j.dib.2016.06.020

**Published:** 2016-06-22

**Authors:** Rongrong Dong, Fahui Li, Shucun Qin, Yi Wang, Yanhong Si, Xuelian Xu, Hua Tian, Lei Zhai, Guangjie Zhang, Yongjun Li, Yawei Zhou, Ying Zhang, Nan Zhang, Shoudong Guo

**Affiliations:** a Key Laboratory of Atherosclerosis in Universities of Shandong Province, Institute of Atherosclerosis, Taishan Medical University, Taian 271000, China; b Department of Chemistry and Chemical Engineering, Weifang University, Weifang 261061, China; c Department of Ophthalmology, Affilated Hospital of Taishan Medical University, Taian 271000, China; d School of Medicine and Pharmacy, Ocean University of China, Qingdao 266003, China

**Keywords:** Immunohistochemistry, LC-MS/MS, Sialic acid, Fluorescent labeling, Imaging

## Abstract

The data presented in this article describe an effect of N-acetylneuraminic acid and/or quercetin on the inflammatory proteins expressions (TNF-α, ICAM-1, VCAM-1 and MOMA-2) and the N-acetylneuraminic acid (NANA) levels of apolipoprotein E-deficient mice that are given a high-fat diet. Protein expression was performed by immunohistochemical imaging and NANA was quantified by liquid chromatography-tandem mass spectrometry (LC-MS/MS) or semi-quantified using Image-Pro Plus software after ligation with fluorescein-5-thiosemicarbazide (FTSC). Further interpretation and discussion could be found at our research article entitled “Exogenous supplement of N-acetylneuraminic acid ameliorates atherosclerosis in apolipoprotein E-deficient mice” (Guo et al., 2016) [1].

**Specifications Table**

**Table:** 

Subject area	*Biology*
More specific subject area	*Immunohistochemistry*
Type of data	*Figure*
How data was acquired	*Microscope, LC-MS/MS*
Data format	*Analysed*
Experimental factors	*Aorta root cryosections (8μm thickness) with the presence of aorta valve cups were prepared by freezing microtome.*
Experimental features	*For immunohistochemistry analysis, aorta roots were dealt with using specific antibodies against* TNF-α, ICAM-1, VCAM-1 and MOMA-2, respectively; For NANA analysis, cryosections were subjected to ligation with FTSC; For LC-MS/MS analysis, NANA was released from aorta by acid hydrolysis and then quantified by LC-MS/MS with precursor to product ion transition of m/z 308.1→86.7.
Data source location	*Institute of atherosclerosis, Taishan Medical University, Tai’an, China.*
Data accessibility	*Data are presented in this article.*

**Value of the data**

• Immunohistochemical data are helpful for explaining the anti-inflammation effect of NANA.
• Data give an example of the influence of exogenous NANA on the endogenous NANA levels of apolipoprotein E-deficient mice.
• The data are helpful for further experiments on staining of NANA at tissue or organ slice *in situ*.
• The data give a basis for further experiments on the underlying functions of NANA.

## 1 Data

**Fig. 1:**
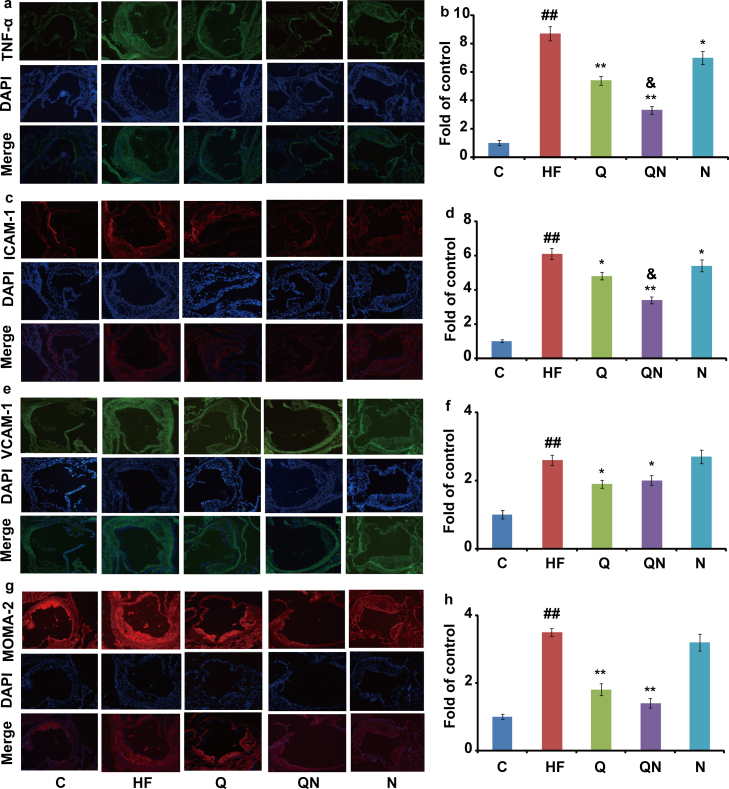
Immunohistochemistry analysis of inflammatory related proteins (*n*=3). (a), (c), (e), (g) Representative images of immunostained aortic cross-sections by specific antibodies against TNF-α, ICAM-1, VCAM-1 and MOMA-2, respectively (20× magnification; blue color signs nuclei, red or green color signs target protein). (b), (d), (f), (h) Densitometric quantification of TNF-α, ICAM-1, VCAM-1 and MOMA-2, respectively. C: chow diet group; HF: high-fat diet model group; Q: quercetin group; QN: quercetin and N-acetylneuraminic acid combination group; N: N-acetylneuraminic acid group. The data are presented as mean±SD. ## means *p*<0.01 *vs* control; * means *p*<0.05 *vs* HF and **means *p*<0.01 *vs* HF; & means *p*<0.05 *vs* quercetin. All of the statistical symbols and the abbreviations are suitable for Fig. 2.

**Fig. 2:**
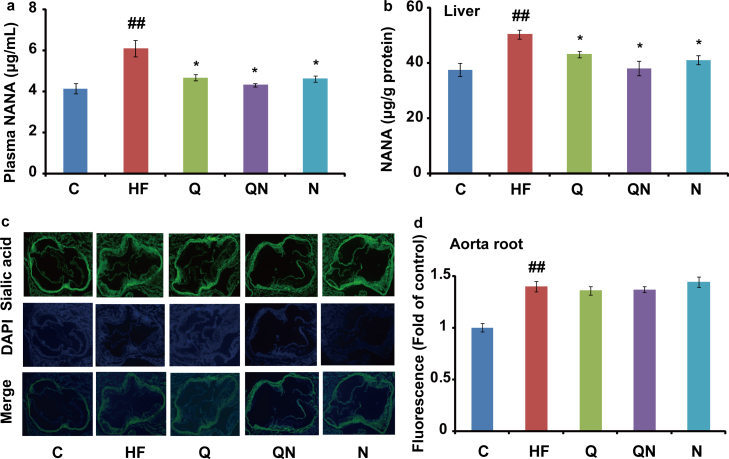
Quantification of NANA by LC-MS/MS and fluorescent staining *in situ*. (a), Plasma levels of NANA quantified by LC-MS/MS (*n*=5). (b). Liver NANA levels quantified by LC-MS/MS (*n*=5). (c) Selective staining NANA in aorta root of *apoE*^*-/-*^ mice by fluorescent reagent. (d), Quantification of NANA in aorta root (*n*=3).

Two figures are presented. Fig. 1 shows the effects of exogenous NANA and/or quercetin on inflammatory proteins expressions as revealed using immunohistochemical imaging. Fig. 2 gives the information of plasma and liver NANA levels induced by a high-fat diet and the influence of NANA and/or quercetin administration.

## 2 Experimental design, materials and methods

### 2.1 Materials

FITC or Cy3 conjugated secondary antibodies were bought from CWBIO (Beijing, China). Detailed information could be found at our research article entitled “Exogenous supplement of N-acetylneuraminic acid ameliorates atherosclerosis in apolipoprotein E-deficient mice” [1].

### 2.2 Instrumentation

NANA analysis was performed with liquid chromatography-tandem mass spectrometry (LC-MS/MS), which was composed of a Shimadzu LC-20AD binary pump, DGU-20A_3_ degasser, SIL-20AC autosampler, and a triple quadrupole mass spectrometer 4000 Q TRAP. High-purity nitrogen for mass spectrometer was produced by nitrogen generator ABN2ZA (Peak Scientific Instruments Ltd.).

### 2.3 Animal harvesting and preparation

For animal grouping and diet, please check the reference [1]. After 8 weeks of treatment, animals were weighed and blood was sampled from the retro-orbital sinus after 12 h fasting. Animals were killed and whole aorta, heart and liver were harvested after perfusion of heart and aorta with ice cold saline. Blood was separated by centrifugation at 1100×*g* for 15 min at 4 °C, and plasma was collected separately. The aortic tree and proximal aorta were separated close to the heart. The proximal aorta, heart and divided liver were embedded with OCT (Thermo). Plasma and the rest tissues were immediately divided and frozen with liquid nitrogen, then kept at −80 °C.

### 2.4 Immunohistochemistry analysis

Serial aortic root cryosections were stained with specific antibodies against TNF-α, ICAM-1, VCAM-1 and MOMA-2. Serial sections were also stained with DAPI. Images were visualized using an Olympus BX51 microscope (Olympus, Tokyo, Japan), and images were captured with a JVC 3-CCD camera (Olympus) and analyzed using Image-Pro Plus software (Version 6.0, Media Cybernetics, LP, USA). The fluorescence intensity of the control group was set at 1, and fluorescence intensity in other groups was determined as a fold of the control.

### 2.5 Quantitative analysis of NANA *in situ*

Aorta root cryosections with the presence of aorta valve cups were dealt with 100 μL of 1 mM NaIO_4_ for selective periodate oxidation of sialic acid at room temperature for 20 min. The oxidation was quenched with 0.5 mL of 1 mM glycerol and washed twice with cold PBS. The cryosections were subjected to ligation with FTSC in PBS buffer (100 μM, pH=7.0) for 40 min [2], then the cryosections were gently washed with PBS 3 times. Nucleus was stained with DAPI according to the manufacturer׳s instruction. Finally, the sections were visualized using an Olympus BX51 microscope (Olympus, Tokyo, Japan), and images were recorded with a JVC 3-CCD camera (Olympus) and analyzed using Image-Pro Plus software (Version 6.0, Media Cybernetics, LP, USA). The fluorescence intensity in the control group was set at 1, and fluorescence intensity in other groups was determined as a fold of the control.

### 2.6 Measurement of NANA by LC-MS/MS

Chromatographic separations were performed with a linear elution using a Waters Symmetry^®^ C18 column (3.5 µm, 2.1 mm i.d.×100 mm) and a Waters C18 guard column (3.5 µm, 2.1 mm i.d.×10 mm). The mobile phase was composed of 2 mM ammonium acetate and 0.1% acetic acid in 5% methanol. Injection volume was 5 µL, and flow rate was 0.4 mL/min. Mass spectrometer was operated in negative ion mode with an ionspray voltage of −4500 V at 500 °C and supplied by auxiliary gas at 55 psi. Nebulizer gas was set at 55 psi, curtain gas at 10 psi and collision gas at medium. Precursor to product ion transition of *m/z* 308.1→86.7 (collision energy −18.0 eV) was used for the selective reaction monitoring of NANA [3]. Quantification was performed with Analyst Software 1.6 (AB SCIEX, Foster, CA, USA).

### 2.7 Data analysis

Detailed analysis methods could be found at our research article entitled “Exogenous supplement of N-acetylneuraminic acid ameliorates atherosclerosis in apolipoprotein E-deficient mice” [1].

## Figures and Tables

**Fig. Transparency document1:**
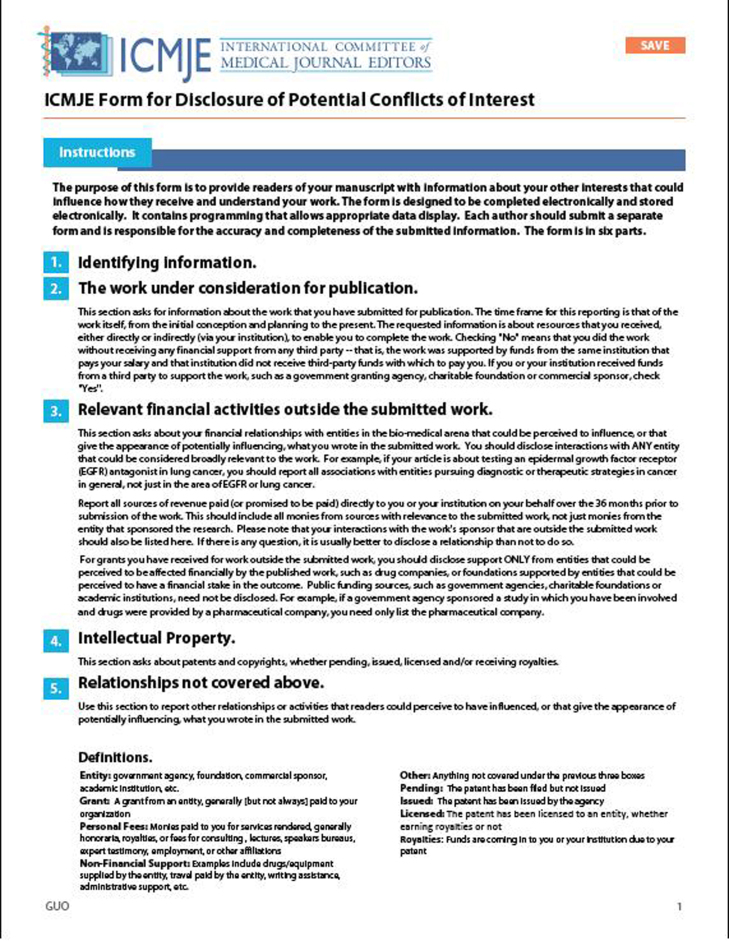


**Fig. Transparency document2:**
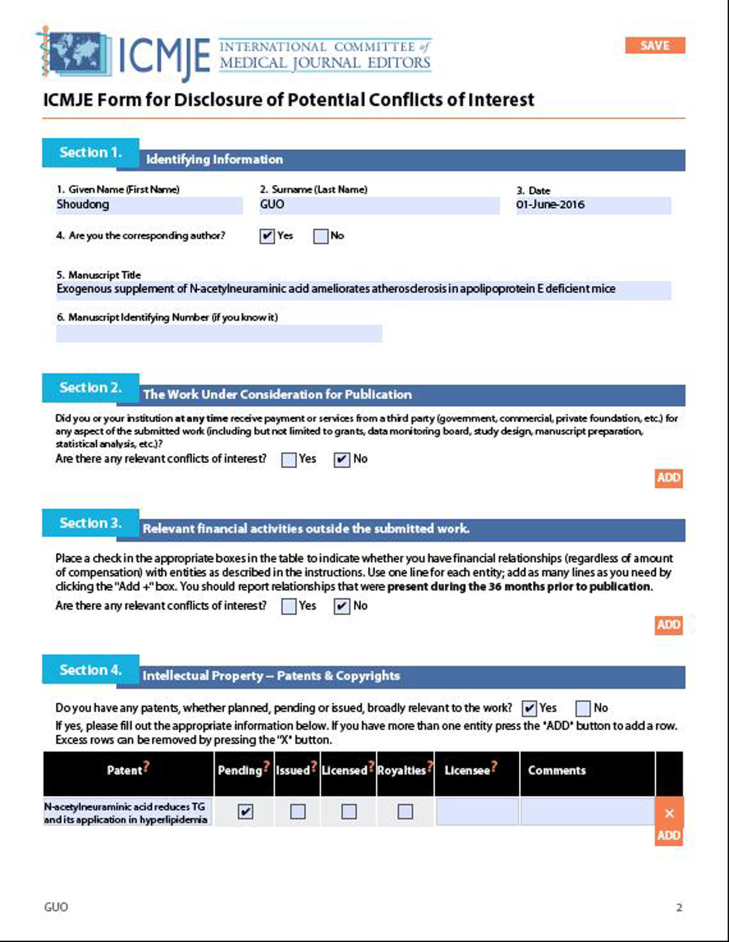


**Fig. Transparency document3:**
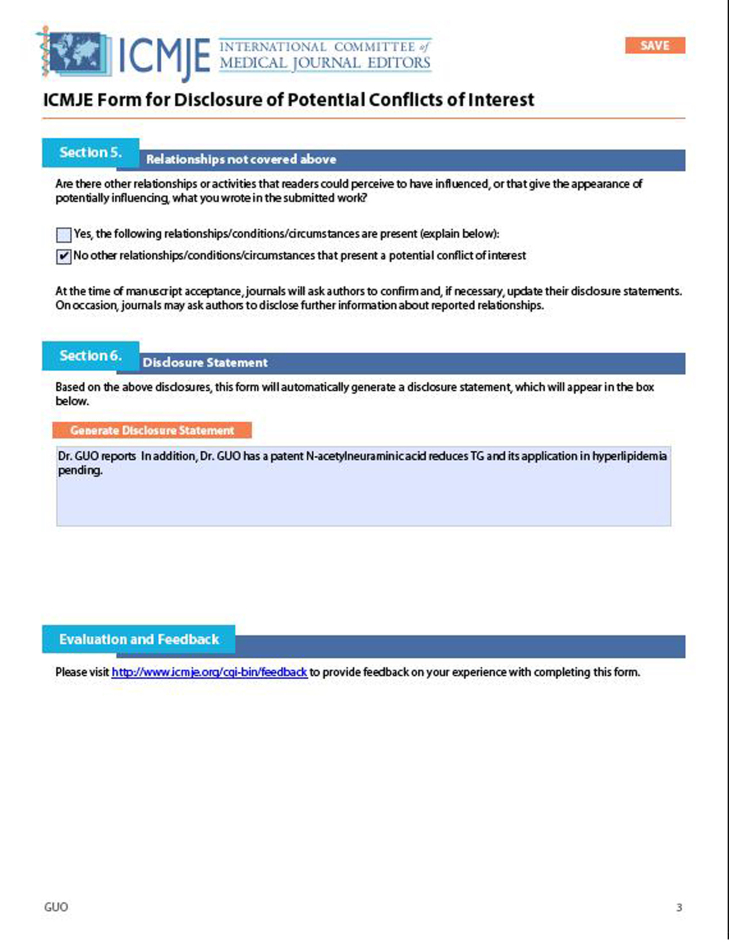


## References

[1] Guo S.D., Tian H., Dong R.R., Yang N.N., Yao S.T., Li Y.J., Zhou Y.W., Si Y.H., Qin S.C. (2016). Exogenous supplement of N-acetylneuraminic acid ameliorates atherosclerosis in apolipoprotein E deficient mice. Atherosclerosis.

[2] Zhang Y., Yuan J., Song J. (2013). An efficient method for selectively imaging and quantifying in situ the expression of sialylated glycoproteins on living cells. Glycobiology.

[3] Guo S.D., Hui S., Yang N.N. (2014). Measurement of sialic acid from lipoproteins and human plasma by liquid chromatography-tandem mass spectrometry. Chin. J. Chromatogr..

